# Enhancing the efficacy of ^131^I therapy in non-toxic multinodular goitre with appropriate use of methimazole: an analysis of randomized controlled study

**DOI:** 10.1007/s12020-019-02100-x

**Published:** 2019-10-04

**Authors:** Piotr Szumowski, Saeid Abdelrazek, Monika Sykała, Małgorzata Mojsak, Łukasz Żukowski, Katarzyna Siewko, Katarzyna Maliszewska, Agnieszka Adamska, Anna Popławska-Kita, Adam Krętowski, Janusz Myśliwiec

**Affiliations:** 1 Department of Nuclear Medicine, Medical University of Bialystok, M. Skłodowskiej-Curie St. 24A, 15-276 Bialystok, Poland; 2 Department of Endocrinology, Diabetology and Internal Medicine, Medical University of Bialystok, M. Skłodowskiej-Curie St. 24A, 15-276 Bialystok, Poland

**Keywords:** Methimazole, Radioiodine, Multinodular goitre, Endogenous thyrotropin

## Abstract

**Purpose:**

It is possible to raise the rate of the uptake of ^131^I in the thyroid gland (RAIU) by increasing the endogenous TSH level through appropriate use of methimazole (MMI) prior to ^131^I therapy. The purpose of this paper is to assess the impact of pre treatment with MMI on the efficacy of ^131^I therapy in non-toxic multinodular goitre (NMG).

**Methods:**

Thirty-one patients with NMG received ^131^I treatment in order to reduce the volume of the thyroid (TVR). Those in group 1 (*n* = 16) were administered 10 mg of methimazole for 6 weeks. Four days after its discontinuation, they received ^131^I. Patients in group 2 (*n* = 15) were given a placebo instead of MMI. The therapeutic activity of ^131^I was constant (800 MBq) and was repeated every 6 months. Treatment was discontinued when TVR reached <40 ml.

**Results:**

In group 1, RAIU increased approximately twofold. Ten patients from group 2 and four patients from group 1 received further doses of ^131^I. The median of time until TVR decreased below 40 ml was 9 months [6–12 months] and 18 months [14–22 months] in group 2. At 2 years after the ^131^I therapy, the occurrence of hypothyroidism did not differ significantly (36% in group 1 and 33% in group2, *p* = 0.074).

**Conclusions:**

Radioiodine treatment of NMG preceded with appropriate application of MMI is efficient thanks to increased RAIU, shorter period of treatment, and lower frequency of ^131^I administration, without an increase in the incidence of post-treatment hypothyroidism.

## Introduction

Apart from surgical methods, radioiodine therapy aimed at reducing goitre size is a basic treatment for non-toxic multinodular goitre (NMG). The efficiency of radioiodine (^131^I) therapy depends on the rate of ^131^I uptake by the thyroid (RAIU). It is widely known that the higher the RAIU, the stronger the effect of ionizing radiation on thyrocytes, which results in greater reduction of goitre size. In NMG, RAIU can be low (sometimes lower than 15–20% after 24 h), which should be attributed primarily to consumption of iodized salt. Decreased RAIU, combined with a large goitre, are the main factors which affect the efficiency of radioiodine therapy in NMG [1–3]. There are a number of methods to increase RAIU, e.g. following a diet low in iodine, lithium, avoiding diuretics. The best of them involves the use of recombinant human TSH (rhTSH) before administering ^131^I. RhTSH boosts RAIU by 100%, or frequently even more, without affecting the half-life of ^131^I in the thyroid (*T*_eff_). In addition, it promotes homogeneous uptake of ^131^I in the thyroid [4, 5]. Routine application of rhTSH in radioiodine therapy of NMG is limited due to the fact that it is currently approved for use only in patients with well-differentiated thyroid cancer, after thyroidectomy, and undergoing radioiodine treatment to ablate the remaining thyroid tissue [6]. Increasing endogenous TSH through appropriately used methimazole (MMI) prior to radioiodine therapy seems to be a viable alternative to rhTSH therapy. The purpose of the paper is to assess the impact of pre treatment with MMI on the efficacy of radioiodine therapy in MNG.

## Materials and methods

### Study population and design

**Fig. 1:**
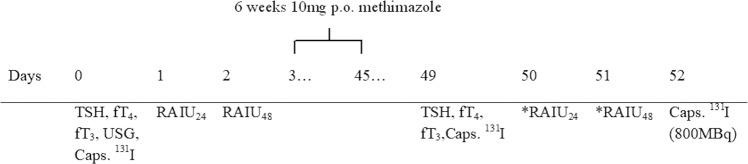
Administration schedule of methimazole for increasing endogenous TSH before radioiodine therapy; (day 0) determination of fT_4_, fT_3_, and TSH levels, USG, administration of ^131^I capsule (4 MBq), (day 1) determination of RAIU_24_, (day 2) determination of RAIU_48_, (days 3–45, 6 weeks) administration of 10 mg of methimazole, (day 49) determination of fT_4_, fT_3_, and TSH levels, administration of ^131^I capsule (4 MBq), (day 50) determination of *RAIU_24_, (day 51) determination of *RAIU_48_, (day 52) administration of ^131^I capsule (800 MBq)

Thirty-one NMG patients received radioiodine therapy with a view to reducing goitre size. Before the treatment, the patients had been randomly divided into two groups using the double blind method (a detailed description of the randomization method is presented below). Subjects in group 1 (*n* = 16) were administered 10 mg of methimazole for 6 weeks. Four days after its discontinuation, they received ^131^I. Subjects in group 2 (*n* = 15) were given a placebo instead of MMI (placebo tablets were identical in size and appearance and they have the same contents as the original methimazole tablets except for the methimazole substance itself, which is not included). Both placebo and methimazole tablets were packed in identical boxes and labelled for the individual patient. At baseline, after randomization and before radioiodine therapy, RAIU was assessed in all the patients. The therapeutic activity of ^131^I was constant: 800 MBq, which is the maximum dose approved for outpatient use in some European countries. If re-treatment was necessary, ^131^I was administered again after an interval of 6 months, in compliance with the accepted protocol (Fig. 1). ^131^I treatment was regarded as complete when thyroid volume reduction (TVR) dropped to <40 ml, which was a clinical endpoint of this study. The secondary endpoints were rate of thyroid volume reduction and impact of RAIU on treatment success. Moreover, before every administration of a therapeutic activity of ^131^I the levels of the thyroid-stimulating hormone (TSH), free thyroxine (fT_4_), triiodothyronine (fT_3_) were measured in all patients. Also, the volumes of the patients’ thyroid glands were determined by means of ultrasound. Follow-up checks of fT_4_, fT_3_, and TSH took place at 3-month intervals until 2 years after the commencement of radioiodine therapy. Ultrasonographic examination was performed every 6 months.

### Inclusion criteria

The inclusion criteria are: NMG (defined as thyroid volume >40 ml, not yet treated), age 18 years or older.

### Exclusion criteria

The exclusion criteria are: major co-morbidity, rendering the participants unlikely to continuously receive the trial intervention, hypersensitivity to MMI, previous treatment with radioactive iodine or surgical.

### Randomization and masking

Allocation to treatment groups was done via remote computerized randomization with minimization to reduce baseline disparities in potential confounding variables between trial interventions.

Patients and clinicians (nuclear medicine specialists), and data analysts were all masked to Assignments. All outcome assessments will be performed blinded and statistical analyses will be performed with the blinding intact.

### Thyroid volume estimation

Thyroid volumes (TV) were measured by means of an ultrasound scanner (LOGIQ S8, GE Healthcare, USA) equipped with a 12 L linear transducer, and calculated based on the equation for an ellipsoid:

$${\rm{TV}} = \frac{4}{3}\pi \times a \times b \times c,$$

where *a*, *b*, *c* are the dimensions of thyroid lobe (length, breadth, and depth).

### RAIU measurements

Radioiodine uptake values after 24 h (RAIU_24 h_) and 48 h (RAIU_48 h_), as well as thyroid scintigraphy examinations were performed with a gamma camera (NuclineTM Th, Mediso, Hungary), using the standard procedure, after per os administration (3 days prior to ^131^I treatment) of a capsule with ^131^I (4 MBq). The effective half-life of ^131^I in the thyroid (*T*_eff._) was calculated on the basis of RAIU_24 h_ and RAIU_48 h_, using a gamma camera.

### Laboratory evaluation

The serum concentrations of the TSH (reference range 0.3–4.0 µIU/ml), fT_4_ (reference range 0.71–1.85 ng/dl), and fT_3_ (reference range 1.45–3.48 pg/mL) were determined by means of an immunoenzymatic method (Microparticle Enzyme Immunoassay-MEIA, Abbott Park, USA).

### Statistical analysis

The statistical analysis of the study results was performed using Statistica 13.1 software (Stat Soft, Tulsa, USA). To compare the two groups of patients in terms of the analysed parameters, the analysis of variance was applied. Changes in thyroid volume and concentrations of hormones were analysed by means of the Friedman test. Multiple regression analysis served to assess the differences between the periods of time required to achieve the desired therapeutic effect in the two studied groups. Kaplan–Meier survival curves and the Cox proportional hazard regression model were used to assess factors behind therapeutic failure (the necessity to administer additional doses of the therapeutic activity of ^131^I). Power calculation was based on the following parameters: 80% power, *α* = 0.05.

## Results

### Baseline characteristics

After randomization, and prior to the radiotherapy, the two groups of patients did not differ statistically in terms of the studied parameters (Table 1).

**Table 1:** Baseline postrandomization characteristics among the two treatment groups

	Group 1 10 mg Methimazol for 1 month (*n* = 16)	Group 2 Placebo (*n* = 15)	*P* value
Age (year)	53.9 ± 8.6	54.3 ± 9.8	0.89
Sex [*n*(%)]			
Male	3 (20)	3 (19)	0.49
Female	12 (80)	13 (81)	
RAIU_24 h_, mean ± SD (%)	29 ± 13	28 ± 13	0.73
RAIU_48 h_, mean ± SD (%)	28 ± 12	27 ± 13	0.78
*T*_eff. (deys)_, mean ± SD	8.03	8.01	0.98
TV (ml), mean ± SD	61.9 ± 19.8	68.3 ± 12.3	0.28
TSH (µIU/ml), mean ± SD	2.1 ± 0.4	2.0 ± 0.4	0.93
fT_4_ (ng/dl), mean ± SD	1.3 ± 0.1	1.1 ± 0.3	0.66
fT_3_ (pg/ml), mean ± SD	2.1 ± 0.3	2.4 ± 0.5	0.85

*n*—number of patients

*fT*_*4*_ free thyroxine, *fT*_*3*_ free triiodothyronine; *TV* thyroid volume; *RA IU*_24 h_ 24-h ^131^I, *RA IU*_48 h_, 48-h ^131^I uptake, *T*_*eff.*_, effective ^131^I half-life in thyroid gland, *TSH*, thyroid-stimulating hormone

### Changes in thyroid parameters after treatment protocol

In patients from group 1, *RAIU_24 h_ and *RAIU_48 h_ (radioiodine uptake in the thyroid post treatment following the protocol as in Fig. 1) considerably increased in comparison with the baseline data from Table 1. This difference (ΔRAIU_24 h_ and ΔRAIU_48 h_) amounts to 36 ± 7 and 31 ± 1%, respectively (slightly more than a twofold rise in the values of both the parameters was noted). Because both *RAIU_24 h_ and *RAIU_48 h_ grew to a similar degree, *T_eff_ (calculated on the basis of the two parameters) did not change in a statistically significant manner: Δ*T*_eff_ = 0.03 ± 0.01, *P* = 0.23. Concentrations of *TSH increased by several times, while *fT4 dropped significantly and *fT3 decreased to a lesser degree (although the levels of all the hormones remained within normal limits), but without marked symptoms of hypothyroidism. In group 2, meanwhile, the differences between all the measured parameters and their baseline values remained statistically insignificant (Table 2).

**Table 2:** Values of parameters after application of protocol and their changes from baseline (see Table 1)

	Group 1	Group 2	*P* value
*RAIU_24 h_/ΔRAIU_24 h_, mean ± SD (%)	65 ± 5/36 ± 7	30 ± 4/2 ± 0.5	*P* = 0.002
*RAIU_48 h_/ΔRAIU_48 h_, mean ± SD (%)	59 ± 3.5/31 ± 1	28 ± 3/1.2 ± 0.5	*P* = 0.0015
*Teff /ΔTeff, mean ± SD (days)	8.04 ± 0.6/0.01 ± 0.01	8.04 ± 0.3/0.3 ± 0.01	*P* = 0.23
*TSH/ΔTSH, mean ± SD	7.9 ± 1.3/5.8 ± 0.6	2.1 ± 0.2/0.1 ± 0.01	*P* = 0.001
*fT4/ΔfT4, mean ± SD	0.7 ± 0.15/0.5 ± 0.2	1.3 ± 0.2/0.2 ± 0.01	*P* = 0.004
*fT3/ΔfT3, mean ± SD	1.5 ± 0.2/0.6 ± 0.2	2.3 ± 0.5/0.1 ± 0.03	*P* = 0.023

Δ—difference between particular parameters in both groups before and after application of accepted protocol, *—parameters after application of accepted protocol

Abbreviations: see Table 1

### Thyroid volume reduction (TVR)

**Fig. 2:**
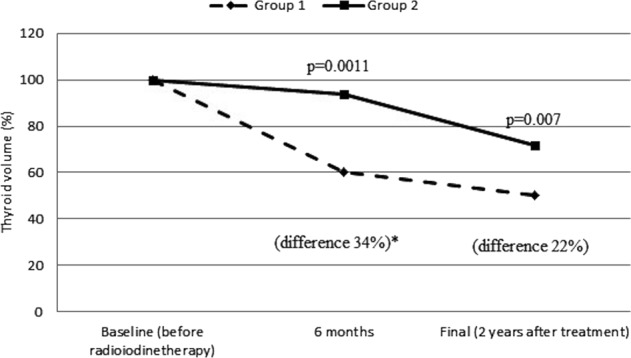
Relative thyroid volume reduction at 6 months and 2 years post treatment with ^131^I alone in group 2 (*n* = 15) and MMI + ^131^I in group 1 (*n* = 16). Asterisk indicates the absolute difference between the two curves at 6 months (34%, *p* = 0.0011) and at 2 years (at the end of the observation period) (22%, *p* = 0.007). Double asterisks indicate all the patients, 4 from group 1 and 10 from group 2 received more than 1 therapeutic activity of ^131^I. Abbreviations: see Table 1

In the course of radioiodine therapy, TVR was significant in both groups, although it was statistically higher in group 1, taking into consideration both measurement periods, i.e., after 6 months and after 2 years (at the end of the observation period). The greatest difference between the two groups was observed after 6 months, when TVR in group 1 was higher by 34% than in group 2. After 2 years, meanwhile, the difference between the groups decreased to 22%. It should be noted that the analysis also comprised those patients (10 from group 2 and 4 from group 1) who had received more than one therapeutic activity of radioiodine. In absolute terms, TV in group 1 were reduced from 80.7 ± 19 ml to 67 ± 17 ml at 6 months and to 41 ± 16 at 2 years post treatment, whereas in group 2 from 82 ± 17 ml to 75 ± 14 ml, and then to 42 ± 12 ml, respectively (Fig. 2).

### Therapeutic failure necessitating re-administration of radioiodine therapy

**Fig. 3:**
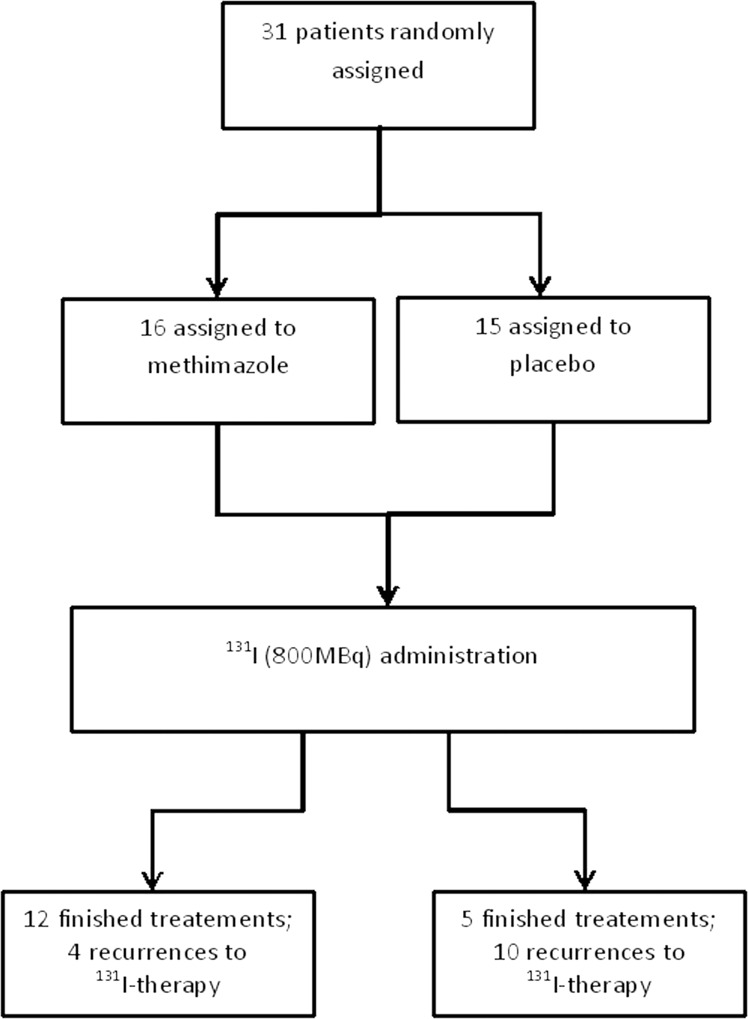
Trial profile

**Fig. 4:**
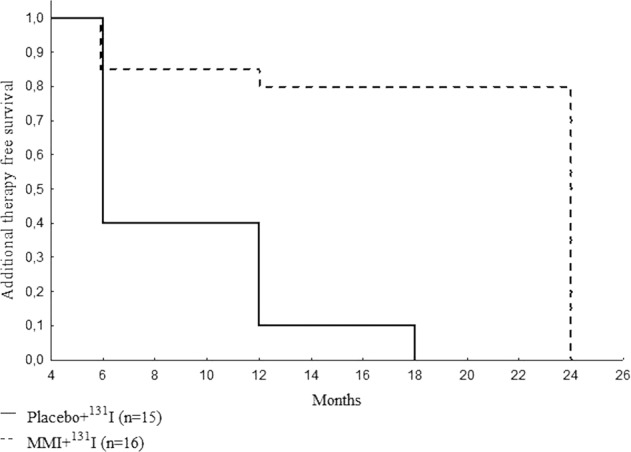
Additional therapy—free survival rate of ^131^I for two randomized groups of patients (Kaplan–Meier estimator)

Ten out of 15 patients who were treated with ^131^I alone received additional doses of ^131^I, as compared to four patients out of the 16 treated with MMI and ^131^I (Fig. 3). In the group which was treated only with therapeutic activity of ^131^I, most of the patients had to receive more than one dose of therapeutic activity of radioiodine and the median of time needed to obtain TVR < 40 ml (see treatment plan in the “Materials and methods” section) in this group was longer, i.e., 18 months [14–22 months], as compared to 9 months [6–12 months] in the group treated with ^131^I and MMI (*p* = 0.017 between the groups) (Fig. 4).

### Probability of receiving additional therapeutic value of ^131^I depending on RAIU_24 h_ and TV

In the Cox regression model containing the mutually dependent variables RAIU_24 h_ and TV, the therapeutic failure rate rose as TV increased and RAIU_24 h_ declined. The hazard ratio for pre-treatment thyroid volume was 1.007, which indicates that an increase in TV by, e.g., every 10 ml means a rise in the rate of therapeutic failure by 7% (1.00710 = 0.07). Meanwhile, an increase in RAIU by every 10% causes a drop in the rate of therapeutic failure by as much as 33% [(1–0.9610)100 = 33%] (Table 3).

**Table 3:** Cox regression estimating the rate of therapeutic failure (requiring additional ^131^I therapy), including TV and RAIU_24 h_

			95% CI for Exp(B)	
Variable	Exp (B)	*P* value	Lower	Upper
TV (ml)	1.007	0.026	1.002	1.014
RAIU_24 h_	0.96	0.01	0.931	0.997

*Exp(B)* Hazard ratio (i.e. the ratio of the hazard rate given a U increase in the covariate to the hazard rate without such an increase), *CI* confidence interval

### Thyroid function

At one year after the administration of ^131^I, five patients from group 1 (31%) and four patients from group 2 (27%) developed hypothyroidism requiring hormonal substitution (*p* = 0.061). The mean doses of thyroxine in both groups also did not differ statistically (*p* = 0.09): 73.6 ± 15 µg/24 h in group 1 and 81.1 ± 17 µg/24 h in group 2.

Thyroid function was assessed 2 years after ^131^I had been given. At the end of that period, hypothyrodism increased (as compared to the results of the assessment made at 1 year post-therapy) by another 5% (to 36%) in group 1 and by 6% (to 33%) in group 2 (*p* = 0.074).

## Discussion

The results of our study show that the TV of patients who had received MMI prior to ^131^I, in compliance with the accepted protocol, decreased significantly more than those of patients treated with ^131^I without having been pre-treated with the thyreostatic. Moreover, the period over which the goitre did not shrink to the desired volume (<40 ml) and the number of administered therapeutic activities of ^131^I were significantly lower in the group of patients treated with the combination of MMI and ^131^I. As far as thyroid function is concerned, receiving MMI had no impact on the incidence hypothyroidism. In view of the above, it can be concluded that using ^131^I in conjunction with MMI increases the efficacy of therapy for NMG. Albino et al., in a 2008 study, were the first authors to draw a similar conclusion, although their sample comprised patients with sub-clinically hypothyroid multinodular goitres and was considerably smaller (9 women) [7]. Two other studies, conducted under the supervision of A. Flores-Rebollar and A. Kyrilli, add, moreover, that the application of MMI prior to a ^131^I therapy can be a safe, easy and acceptable method to increase TSH concentrations, representing a valid alternative to the widely used rhTSH. This is because both the methods of stimulating RAIU cause an approximately twofold increase in its value [8, 9]. Our research shows that the applied adjuvant therapy with MMI caused an over twofold rise not only in RAIU_24 h_ but also in RAIU_48 h_: 2.24 and 2.11, respectively. The comparable rise in both RAIU values had no impact on *T*_eff._ (there was no statistical difference in either of the groups; group 1: *T*_eff._ = 8.04 ± 0.6, group 2: *T*_eff._ = 8.04 ± 0.3; *p* = 0.23), which consequently does not lead to a shortening of the exposure of thyroid tissue to radiation [10, 11].

In other words, the increase in the absorbed dose of ^131^I radiation in the thyroid directly depends on the rise in RAIU, irrespective of the influence of *T*_eff._ (as *T*_eff._ is unchanging). This is the characteristic not only of using MMI but also rhTSH before ^131^I therapy [12]. In both treatment methods, stimulation of RAIU by increasing TSH takes so long that *T*_eff._ equals the physical half-life of ^131^I (*T*_phys_. = 8.04 days).

The same authors also address important aspects of radiological protection of patients undergoing treatment. Pre-stimulation with rhTSH reduces the dose absorbed by the urinary bladder in comparison with conventional radioiodine therapy. This is associated with reduced renal elimination of ^131^I, thanks to higher RAIU [12–14]. Analogous conclusions can be reached as regards appropriate dosing of methimazole before ^131^I-therapy, particularly in NMG, where this is the most clearly observable (because of frequently low RAIU prior to treatment).

As is known, significant TVR (in this study: up to < 40 ml) in possibly the shortest time is the main measure of efficacy in the treatment of NMG. In our study, the reduction in TV after ^131^I therapy over the period of 1 year and at the end of the study period was significantly higher in the group of patients treated with MMI than in the group treated with ^131^I alone (the difference between the groups amounted to 34% and 22%, respectively). Similar results were obtained by authors led by Soren Fast, with the difference that they used rhTSH to increase serum concentrations of TSH. In patients who had received rhTSH, TVR was greater by 16% at one year after ^131^I therapy and by 14% at 3 years [15]. Conversely to our results, the aforementioned authors found that the time necessary to obtain the desired reduction in the TV of the patients who had undergone stimulation of RAIU was longer (73 months) than in the case of those who had been treated with ^131^I alone. They explain that ^131^I was administered more frequently in the group which had not received rhTSH. Our Cox regression analysis model demonstrates that the period of ^131^I treatment for NMG can be shortened as a result of appropriate use of MMI (causing a rise in RAIU). We have shown that increased RAIU significantly diminishes the risk of therapeutic failure (e.g. every 10% rise in RAIU causes a drop in the rate of therapeutic failure by as much as 33%).

In spite of the substantial improvement of the efficacy of ^131^I-treatment in conjunction with MMI in comparison to standard radioactive iodine therapy, the occurrence of hypothyroidism did not increase. Admittedly, this result may be affected by an estimation error due to the relatively small patient sample (31 persons). Nevertheless, other authors reached similar conclusions, even though in their studies, the rise in RAIU was achieved through administration of rhTSH, instead of MMI [16, 17].

The only limitation of ^131^I- therapy with MMI is hypersensitivity to MMI in some patients, which can be reduced to a minimum by short period of the intake of this drug.

## Conclusions

^131^I therapy for NMG preceded with appropriately dosed MMI increases the efficacy of treatment. Several factors are involved: higher RAIU, shortened period of treatment, lower frequency of ^131^I administration in order to achieve satisfying level of TV, smaller dose of radiation absorbed by the other organs, without increasing the risk of posttherapeutic hypothyroidism. The method can be an alternative to ^131^I therapy for NMG with adjuvant rhTSH.
